# The processing of faces across non-rigid facial transformation develops at 7 month of age: a fNIRS-adaptation study

**DOI:** 10.1186/1471-2202-15-81

**Published:** 2014-06-26

**Authors:** Megumi Kobayashi, Yumiko Otsuka, So Kanazawa, Masami K Yamaguchi, Ryusuke Kakigi

**Affiliations:** 1 Department of Integrative Physiology, National Institute for Physiological Sciences, 38 Nishigo-Naka, Myodaiji, Okazaki, Aichi 444-8585, Japan; 2 Japan Society for the Promotion of Science, 5-3-1, Koujimachi, Chiyoda, Tokyo 102-0083, Japan; 3 School of Psychology, The University of Sydney, Sydney, NSW 2006, Australia; 4 Department of Psychology, Japan Women’s University, 1-1-1, Nishi-ikuta, Tama, Kawasaki, Kanagawa 214-8565, Japan; 5 Department of Psychology, Chuo University, 742-1, Higashi-nakano, Hachioji, Tokyo 192-0393, Japan

## Abstract

**Background:**

Using near-infrared spectroscopy (NIRS), our previous neural adaptation studies found that infants’ bilateral temporal regions process facial identity (FiHN 5:153, 2011). In addition, we revealed that size-invariant processing of facial identity develops by 5 months of age (NR 23:984-988, 2012), while view-invariant processing develops around 7 months of age (FiHN 5:153, 2011). The aim in the current study was to examine whether infants’ brains process facial identity across the non-rigid transformation of facial features by using the neural adaptation paradigm. We used NIRS to compare hemodynamic changes in the bilateral temporal areas of 5- to 6-month-olds and 7- to 8-month-olds during presentations of an identical face and of different faces.

**Results:**

We found that (1) the oxyhemoglobin concentration around the T5 and T6 positions increased significantly during the presentation of different faces only in 7- to 8-month-olds and (2) 7- to 8-month-olds, but not 5- to 6-month-olds, showed attenuation in these channels to the presentation of the same face rather than to the presentation of different faces, regardless of non-rigid changes in facial features.

**Conclusions:**

Our results suggest that the processing of facial identity with non-rigid facial transformation develops around 7 months after birth.

## Background

Using near-infrared spectroscopy (NIRS), many recent studies have explored brain activities underlying face recognition in infants [1-7]. These studies have revealed infants’ brain activation patterns during the presentation of upright and inverted faces [1], facial expressions [2], frontal and profile views [3], mother’s face and unfamiliar female faces [4,5], Arcimboldo images [6], and directed and averted gaze [7]. These studies suggest that face stimuli evoke cortical activation in infants’ bilateral temporal areas.

To examine which facial information is processed in infants’ temporal areas, we have applied the neural adaptation technique to infant NIRS measurement [8,9]. The technique of neural-adaptation has been established in adult neuroimaging studies, especially functional magnetic resonance imaging (fMRI), as a tool for investigating the functional properties and representations processed in particular neural populations. Neural-adaptation refers to the attenuation of brain activation by the repeated presentation of an identical stimulus compared to that of different stimuli, e.g. [10,11]. By measuring the recovery from adaptation when some stimulus property is altered, the nature of representation in a specific cortical area can be assessed. At first, we examined whether lower hemodynamic responses occurred for an identical face than for different faces [8]. Results showed that infants aged 5 to 8 months showed an attenuated hemodynamic response (adaptation) to the identical face. We revealed that such adaptation is also observed in 5- to 8-month-olds when the sizes of the faces were changed [9]. In contrast, when the faces were presented from multiple viewpoints, only 7- to 8-month-olds, but not 5- to 6-month-olds, showed the adaptation [8]. These previous studies suggest that (1) infants’ temporal areas process facial identity in a size-invariant manner at 5 months of age, (2) the ability to process facial identity in a view-invariant manner develops around 7 months of age.

In the present study, we investigated image-invariant recognition of faces by infants for face changes associated with non-rigid facial movement. Facial movement can be categorized into two different types: rigid movement and non-rigid movement. Rigid movement is the rotation of the head, which provides a different view of the face [12]. Non-rigid motion is the movement of the internal facial features, which provides visual information relating to facial expressions, eye gaze changes and speech [13]. As noted above, our previous NIRS-adaptation study already examined the processing of facial identity across changes with rigid movement and revealed that the ability to process facial identity develops around 7 months of age [8]. Here, we focused on the processing of facial identity in 5- to 8-month-olds’ temporal areas across changes of facial features associated with non-rigid movement.

Ichikawa et al. [14] created point-light displays (PLDs) depicting faces with dynamic facial expression. They measured infants’ brain activity as the infants viewed upright and inverted versions of the PLDs. They found increased brain activation for the upright PLDs in 7- to 8-month-olds’ temporal areas, suggesting not only that biological motion enhanced infants’ hemodynamic response to faces, but also that the infants recognized the faces from only the facial movement depicted by the PLDs. Their results can be interpreted as showing that infants aged around 7 months process facial expression through non-rigid facial movement. We used NIRS to investigate whether 5- to 8-month-old infants process facial identity from faces across non-rigid facial change. We postulated that if infants could process the invariance of facial identity across non-rigid facial change, they would show an attenuation of oxy-Hb concentration (adaptation) to the presentation of an identical face rather than different faces, even across non-rigid facial change as in our previous studies [8,9].

## Results

We obtained hemodynamic responses from twelve 5- to 6-month-olds and twelve 7- to 8-month-olds who looked at the stimuli for more than three trials in both the *same-face* and *different-face* conditions. The mean number of valid trials was 4.9 (*SD* = 1.4) for the *different-face* condition and 4.8 (*SD* = 1.7) for the *same-face* condition in 5- to 6-month-olds, and 5.2 (*SD* = 1.5) for the *different-face* condition and 5.5 (*SD* = 2.2) for the *same-face* condition for 7- to 8-month-olds. As we could successfully establish a sufficient contact between the optical fibers and the skull for all 24 channels for all twenty-four infants mentioned above, data from all channels were included in the analysis. To investigate the possibility that infants had habituated to the faces through repeated presentation across trials during the course of experiment, we compared infants’ looking time between the first and the last trial for both conditions by an ANOVA with three factors: age of groups (5- to 6-month-olds vs. 7- to 8-month-olds), condition (*same-face* vs. *different-face*), and trial (first vs. last trial). As a result, we did not find any main effects (age *F*(1, 22) = 1.07, *p* = .31; condition *F*(1, 22) = 1.88, *p* = .18; trial *F*(1, 22) = 0,96, *p* = .34) or interaction (age × condition *F*(1, 22) = 0.02, *p* = .88; age × trial *F*(1, 22) = 0.98, *p* = .34; condition × trial *F*(1, 22) = 1.72, *p* = .20; age × condition × trial *F*(1, 22) = 0.19, *p* = .67), suggesting no significant differences in looking time, which argue against the possibility that infants had habituated to the faces during this experiment.

**Figure 1:**
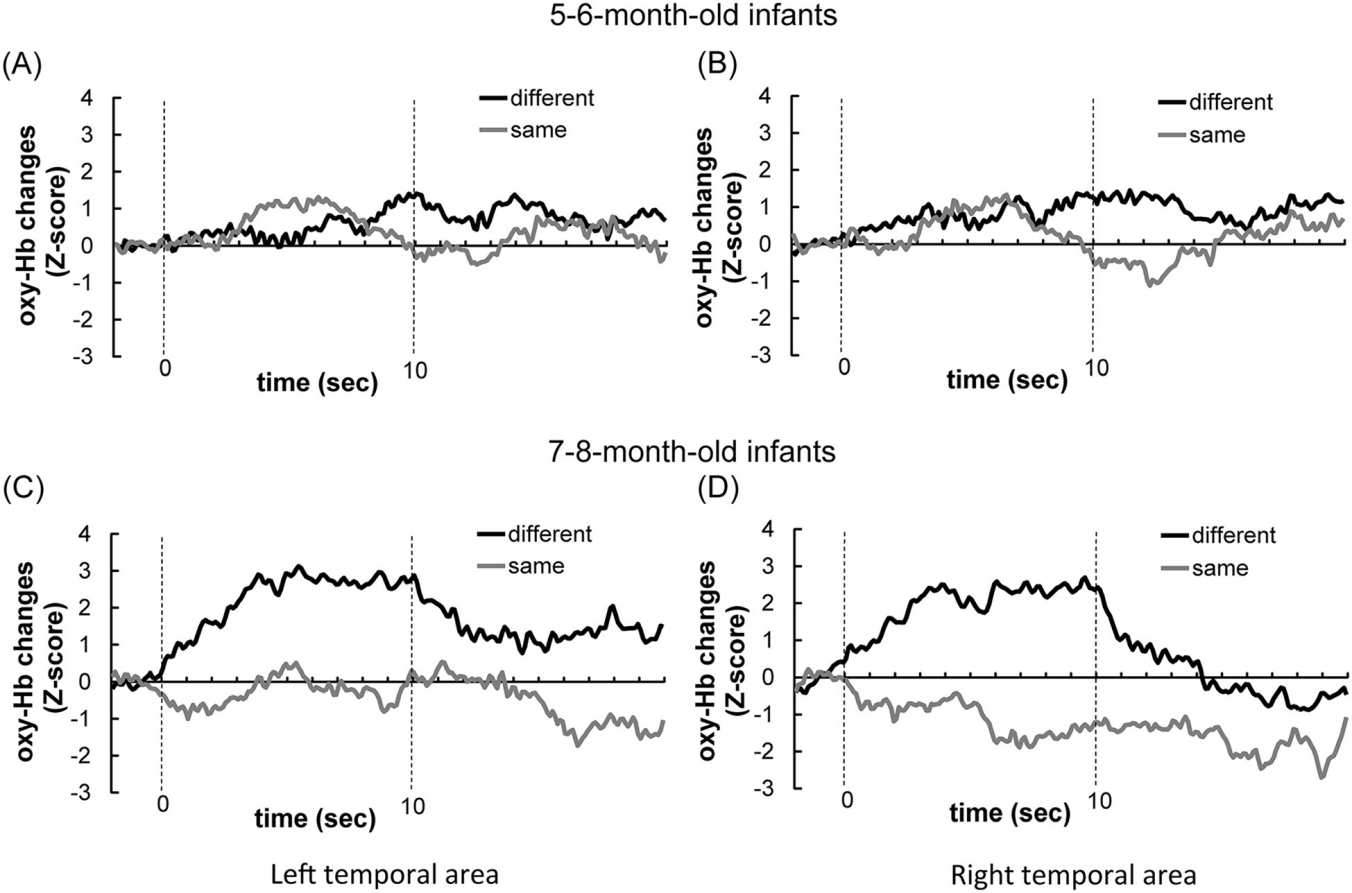
**The time courses of oxy-Hb concentration in 5- to 6-month-olds (A and B) and 7- to 8-month-olds (C and D).** The time course of the oxy-Hb concentration in the bilateral temporal areas obtained from the 12 channels for each hemisphere under the *different-face* condition (the black line) and the *same-face* condition (the gray line) in 5- to 6-month-olds **(A and B)** and 7- to 8-month-olds **(C and D)**. Zero on the horizontal axis represents the beginning of the test period, and 10 represent the end of the test period.

Figure 1 shows the time course of the average oxy-Hb concentration in the bilateral temporal areas obtained from 12 channels for each hemisphere under the *different-face* condition (the black line) and *same-face* condition (the gray line). In 7- to 8-month-olds, increases of oxy-Hb concentration in the *different-face* condition occurred at about 3 s after the beginning of the test trial, and the decrease occurred after the end of the test trial (Figure 1C and D). In contrast to the oxy-Hb changes, the deoxy-Hb changes in both temporal areas increased in the *same-face* condition rather than the *different-face* condition. 5- to 6-month-olds did not show any changes in either condition compared to the baseline (Figure 1A and B).

### Developmental changes in facial identity processing across non-rigid facial changes?

**Figure 2:**
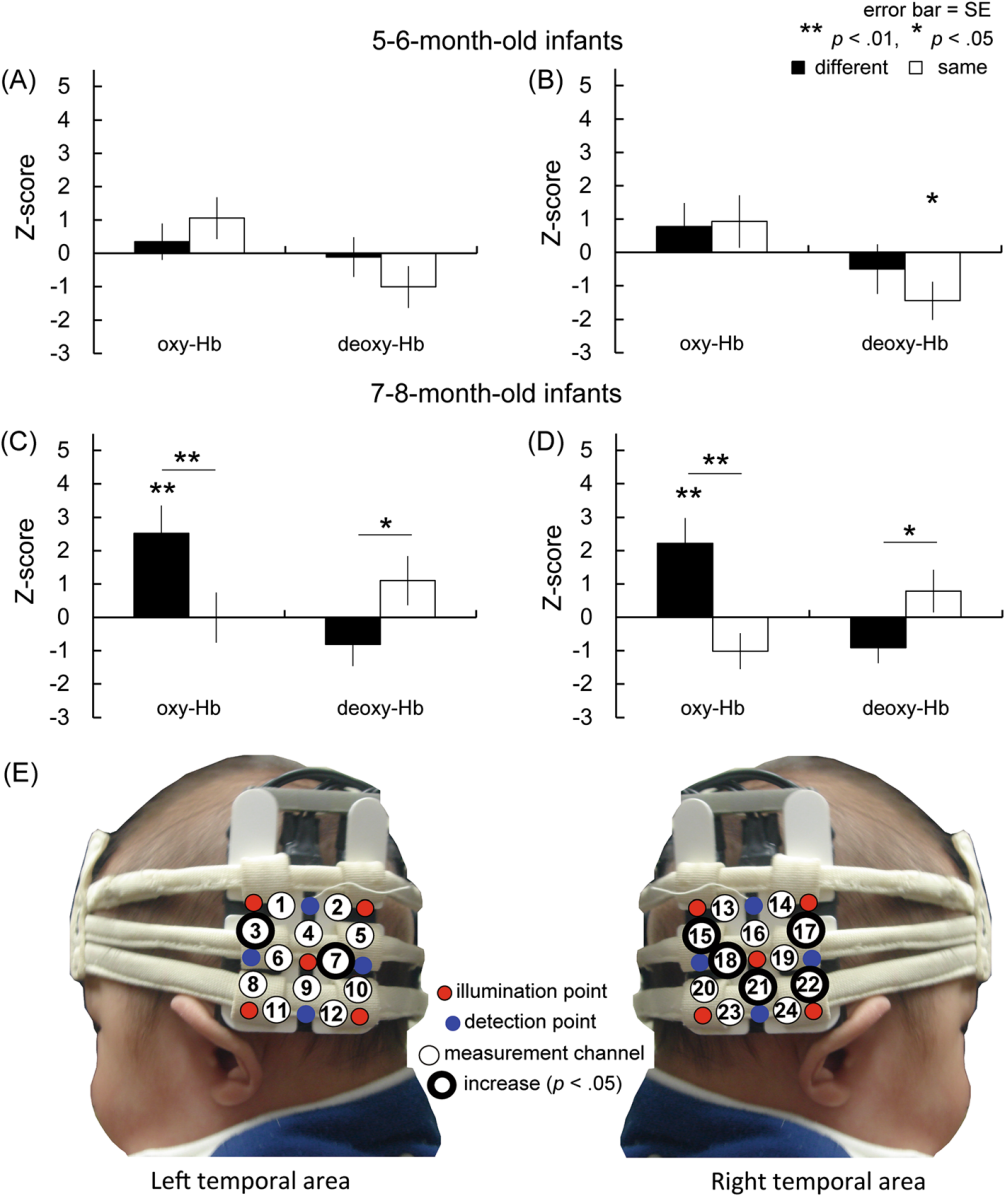
**Mean Z-score of oxy- and deoxy-Hb (A-D) and the channels that showed significant increases of oxy-Hb (E). (A-D)** The black bar represents the mean Z-score of the *different-face* condition, and the white bar represents that of the *same-face* condition. For 5- to 6-month-olds **(A and B)**, deoxy-Hb in the *same-face* condition significantly decreased in the right temporal area. At 7- to 8-month-olds **(C and D)**, oxy-Hb concentration in both temporal areas significantly increased compared to the baseline (0). Furthermore, oxy-Hb and deoxy-Hb in bilateral temporal areas significantly differed between the *same-face* and *different-face* condition. **(E)** The channels showed significant increases of oxy-Hb in the *different-face* condition compared to the baseline period in 7- to 8-month-olds (two-tailed *t*-test, *p* < .05). The significant *p*-value α was determined on the basis of the method of controlling the false discovery rate. In these channels, oxy-Hb concentration differed significantly between the *same-face* condition and the *different-face* condition. (The infant’s parents provided written informed consent for publication of this photograph.).

A repeated-measures ANOVA with age (5- to 6-month-olds vs. 7- to 8-month-olds) as a between-subject factor, and condition (*same-face* vs. *different-face*) and measurement area (right vs. left) as the within-subject factor, was separately performed on the average Z-score of oxy- and deoxy-Hb from 3 s to 7 s of the test trial in each temporal area (Figure 2A-D). This analysis revealed a significant interaction between age and condition for oxy-Hb (*F*(1,22) = 6.26, *p* < .05, η^2^ = .53). According to multiple comparisons, the oxy-Hb concentration in the *different-face* condition was significantly higher than that of the *same-face* condition for the older group (*F*(1,11) = 9.47, *p* < .01, η^2^ = .92). No main effect or other interaction reached statistical significance (main effect: age *p* = .82, condition *p* = .07, hemisphere *p* = .13; interaction: between age and hemisphere *p* = .06, between condition and hemisphere *p* = .25, between 3 factors *p* = .88). For deoxy-Hb, the interaction between age and condition was also significant (*F*(1,22) = 5.25, *p* < .05, η^2^ = .48). Multiple comparisons revealed that deoxy-Hb in the 7- to 8-month-olds significantly differed between the two conditions (*F*(1, 22) = 4.61, *p* < .05, η^2^ = .45). No main effect or other interaction reached statistical significance (main effect: age *p* = .17, condition *p* = .46, hemisphere *p* = .26; interaction: between age and hemisphere *p* = .71, between condition and hemisphere *p* = .76, between 3 factors *p* = .85).

### The channels showing increased hemodynamic responses to faces

To examine the possibility that there was differential activity for the presentation of faces compared to the baseline, we performed a two-tailed one-sample *t*-test on the Z-scores against a chance level of 0 for each age group separately. The analysis revealed that in 7- to 8-month-olds, the concentration of oxy-Hb increased significantly in both the left and right temporal areas during the *different-face* condition (left: *t*(11) = 2.52, *p* < .05, *r* = .61, 95% CI [0.43 4.61], right: *t*(11) = 2.95, *p* < .05, *r* = .67, 95% CI [0.34 4.10]), but not during the *same-face* condition (*p* > .10). In contrast to the 7- to 8-month-olds, no significant change in the concentration of oxy-Hb was found during the *different-face* or *same-face* conditions in the 5- to 6-month-olds (*p* > .10). For deoxy-Hb changes, deoxy-Hb in the 5- to 6-month-olds significantly decreased only in the *same-face* condition in the right temporal area (*t*(11) = -2.56, *p* < .05, *r* = .61, 95% CI [-2.86 -0.03]).

In the 7- to 8-month-olds, 7 channels around the T5 and T6 positions showed significant increases in oxy-Hb in the *different-face* condition from the object baseline, but not in the *same-face* condition (*p* < .05, two-tailed one-sample *t*-test, FDR corrected; Figure 2E). In contrast, no significant change was found in either the *same-face* or the *different-face* condition in 5- to 6-month-olds (*p* > .10). These seven channels (ch. 3, 7, 15, 17, 18, 21, and 22) were selected as COI (Channels of Interest). A repeated-measure ANOVA with two factors, condition (*same-face* vs. *different-face*) and channel (ch. 3, 7, 15, 17, 18, 21, and 22), was performed on the data of oxy-, deoxy-Hb in the COI region. This analysis revealed a significant effect of condition only for oxy-Hb (*F*(1,11) = 14.89, *p* < .01, η^2^ = 1.16). The main effect of channel (*p* = .18) and interaction (*p* = .09) were not significant.

## Discussion

The current study used the NIRS-adaptation paradigm to investigate the development of infants’ processing of facial identity across non-rigid facial transformation. We compared brain activation in the bilateral temporal areas between the presentation of multiple faces (*different-face* condition) and an identical face (*same-face* condition), both transformed non-rigidly by showing different facial gestures. We found that despite the non-rigid facial change, hemoglobin changes in channels around the T5 and T6 positions were significantly lower for the *same-face* condition than for the *different-face* condition only in 7- to 8-momth-olds.

The current study suggests that infants’ processing of facial identity across non-rigid facial changes develops around 7 months of age. The pattern of results we obtained in infants’ bilateral temporal region is consistent with those obtained in the fusiform face area (FFA) of adults in previous studies [15,16]. However, this does not necessarily imply that our results reflect activity in infants’ FFA. Given that NIRS can record the hemodynamic responses only in the superficial layers of the cortex, the adaptation we showed in the present study might reflect activation not in the FFA, but in the superior temporal sulcus (STS) [8,9].

Previous behavioral study has reported that 3- to 4-month-old infants could learn a facial identity from a movie showing non-rigid facial motion and recognize the face in expressions that differed from the learned facial image [17]. For infants’ sensitivity to non-rigid facial transformation, ERP studies reported the ability to discriminate between an angry facial expression and a neutral expression in infants aged around 7 months [18,19]. Infants’ sensitivity to rigid changes of face have been also reported in behavioral [20] and neuroimaging studies [3]. Previous studies reported that infants’ ability to recognize face across different views was shown even in newborn infants [21] and becomes more robust at around 8 months after birth [20]. Consistent with these behavioural findings, the same activity between frontal and side-view faces was reported in 8-month-old infants, not but in 5-month-old infants [3]. Taken together, the results of these previous studies and the present study suggest that 7- to 8-month-olds develop the ability to process not only information of facial transformation, but also facial identity across the facial transformation.

The stimuli in the current study consisted of images from only five individual faces. As the images of five faces were repeatedly shown during the experiment, this might have caused attenuation in the hemodynamic responses over the course of the trials. To examine this possibility, we compared the oxy-Hb changes in the *same-face* and *different-face* conditions between the first and last trials by an ANOVA with three factors: hemisphere (right vs. left), condition (*same-face* vs. *different-face*) and trial (first vs. last trial). As a result, we found no main effect and any interaction in either 5- to 6-month-olds (main effect: hemisphere *p* = .23, condition *p* = .11, trial *p* = .52; interaction: hemisphere × condition *p* = .64, hemisphere × trial *p* = .74, condition × trial *p* = .54, hemisphere × condition × trial *p* = .89) or 7- to 8-month-olds (main effect: hemisphere *p* = .27, condition *p* = .84, trial *p* = .72; interaction: hemisphere × condition, *p* = .14, hemisphere × trial *p* = .94, condition × trial *p* = .74, hemisphere × condition × trial *p* = .41). This result suggests that increased oxy-Hb concentration for the *different-face* condition occurred throughout the experiment in 7- to 8-month-olds, while 5- to 6-month-olds showed no changes throughout the experiment.

In the current study, 5- to 6-month-olds did not show increases in oxy-Hb concentration in either the *different-face* or *same-face* condition. Considering previous behavioral findings that even 5-month-olds had the ability to process changes of the internal features of face [22,23], we predicted that 5- to 6-month-olds would show increases for both conditions. The pattern of younger infants’ hemodynamic responses in this study suggests differences between behavioral responses and neuroimaging responses. Although 5-month-olds can process changes of the internal features of face, this process might not be reflected in neural responses [24]. The 5- to 6-month-olds’ hemodynamic responses in this study are in accordance with those of our previous adaptation study examining view-invariant face processing [8].

Inconsistent results between this study and previous behavioral studies might also result from a difference in learning time. In this study, infants were required to recognize faces without a prior learning phase. In contrast, previous behavioral studies [22,23] used an infant-controlled habituation procedure in which infants were given considerable time to learn the faces before the recognition test. Taken together, these studies suggest that infants aged 5 to 6 months needed sufficient learning time to process the internal features of faces. Our results with the 5- to 6-month-olds did not necessarily show that 5- to 6-month-olds lack the ability to process changes in internal facial features.

A series of our adaptation studies (the present study and Kobayashi et al. [8,9]) have suggested that infants’ temporal areas are involved in the processing of facial identity. The size of adaptation, as indexed by the difference in activity between *different-face* and *same-face* conditions, was similar to that of our previous study for rigid facial change (Left *M* = 2.01, *SD* = 2.34, Right *M* = 1.79, *SD* = 2.07) [8] and to that of the present study for non-rigid facial change (Left *M* = 2.52, *SD* = 4.16, Right *M* = 3.24, *SD* = 3.59). A two-way ANOVA on the size of adaptation with hemisphere (Left vs. Right) and condition (rigid change vs. non-rigid change) revealed no difference between the two studies (main effect of condition *p* = .42, hemisphere *p* = .59, interaction *p* = .31). Thus, our results for small adaptation in the *different-face* condition did not stem from the variation of faces, e.g., the shape or location of facial features. We also have revealed that 1) the processing of facial identity invariant to size change emerges before 5 months of age, and 2) the processing of facial identity across the change, along with rigid and non-rigid facial movement, develops around 7 months of age. By applying the neural adaptation paradigm established in fMRI study to NIRS measurement in infancy, we obtained the first evidence that the processing of facial identity in the bilateral temporal areas develops from a low level to a higher level by 8 months of age.

## Conclusions

Using the NIRS-adaptation paradigm, the current study investigated the development of facial identity processing in 5- to 8-month-old infants for non-rigid transforming facial features. We focused on whether attenuated hemodynamic response (adaptation) occurred in infants’ temporal areas for an identical face rather than for different faces. As predicted, 7- to 8-month-old infants showed the adaptation for the presentation of the same face, while 5- to 6-month-old infants did not. This result suggests that the ability to process facial identity across non-rigid facial changes develops around 7 months after birth.

## Methods

### Participants

The final sample of the present study consisted of twenty-four full-term and healthy Japanese infants, twelve 5- to 6-month-old infants (7 boys, 5 girls, *M* age = 165.3 days, age range: 142-192 days) and twelve 7- to 8-month-old infants (6 boys, 6 girls, *M* age = 231.8 days, age range: 208-253 days). An additional seven infants were excluded because of an insufficient number of available trials (less than 3 trials for the *same-face* and *different-face* condition, respectively), crying, or motion artifacts. This study was approved by the Ethical Committee of Chuo University (2012-8) and the National Institute for Physiological Sciences (19), and written informed consent was obtained from the parents of the infant participants. The experiments were conducted according to the Declaration of Helsinki.

### Stimuli

**Figure 3:**
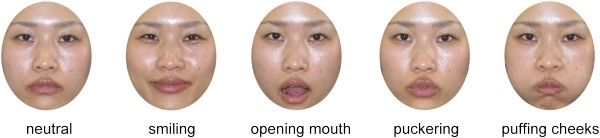
**Example of face stimuli.** These images contain the following facial gestures: neutral, smiling, opening mouth, puckering, and puffing cheeks. (The individual provided written informed consent for publication of these images.).

The stimuli for the test period consisted of 25 full-color photographs of five different Japanese adult female faces in a frontal viewpoint. There were five images of each female with different facial gestures: neutral, smiling, opening mouth, puckering, and puffing cheeks (Figure 3). The RGB content of the facial images (histogram for each channel) was equated across the five female faces using SHINE Toolbox (Université de Montréal, Montréal, Québec, Canada; http://www.mapageweb.umontreal.ca/gosselif/shine/) [25]. The stimuli for the baseline period consisted of full-color photo images of the five vegetables used in our previous studies [1-4,6,8,9,26]. The sizes of the stimuli were approximately 17.5° × 21° in visual angle for the faces, and 16.8° × 16.8° for the vegetables.

There was a baseline period and a test period. Images were shown in a flashing manner during both the baseline period and the test period. During the test period, each facial image flashed for a duration of 800 ms, and a 200 ms ISI was filled with a fixation point (a small red cross). The flashing of facial images was repeated for the duration of 10 s in each trial. There were two conditions for the test period: the *same-face* condition and the *different-face* condition. In both conditions, the faces were shown with different facial gestures, with all five facial gestures shown in random order within each trial. In the *different-face* condition, the identity of faces also varied across the flash within a trial with all five female faces shown in random order in each trial. The identity of the face remained constant within a trial in the *same-face* condition. The order of presentation of the five different gestures was randomly determined for each trial. The order of the identity for the *different-face* condition was randomly determined for each infant. The identity of the face in the *same-face* condition differed trial by trial without repeat until all five faces were presented. The order of presentation of the five faces was randomized for each infant. The *same-face* condition and *different-face* condition were presented at alternating trials. The order of the two conditions was counterbalanced across infants.

As in our previous studies [8,9], the five vegetables were shown in a random order at a rate of 0.5 Hz during the baseline period. As with the test period, each vegetable’s image flashed for a duration of 800 ms, and a 200 ms ISI was filled with a fixation point (a small red cross). Each trial followed a baseline period of at least 10 s. The duration of the baseline period was controlled by the experimenter. The results obtained from viewing the objects were used as the baseline.

To draw and keep the attention of the infants, both the face stimuli and the vegetables were accompanied by a beeping sound presented at 1 Hz. Two different sounds were used for the face stimuli and the vegetables, and the same sound was used in both the *same-face* and *different-face* conditions. The relationship between the sounds and the visual stimuli was counterbalanced across infants.

### Procedure

Each infant was tested while sitting on the experimenter’s lap and facing a 22-inch CRT monitor 40 cm away. The infants watched the stimuli passively while their brain activity was measured, and the trials were repeated as long as infants were willing to look at the stimulus display. The participants’ behavior was videotaped during the experiment.

### Recording

We used a HITACHI ETG-4000 device system (Hitachi Medical, Chiba Japan), which can record NIRS from 24 channels simultaneously, with 12 channels for the right temporal area, and 12 for the left. The instrument generates two wave lengths of NIR (695 and 830 nm). The HITACHI ETG-4000 measured the time courses of the levels of oxyhemoglobin (oxy-Hb), deoxyhemoglobin (deoxy-Hb), and the sum (total hemoglobin, total-Hb) at 24 channels with 0.1-s time resolution. We used a pair of probes, each containing nine optical fibers (3 × 3 arrays). Of the nine fibers, five were emitters, and four were detectors. The optical fibers of each probe were kept in place with a soft silicon holder. The distance between the emitters and detectors was set at 2 cm because each pair of adjacent emitting and detecting fibers defined a single measurement channel, which allowed for the measurement of oxy-Hb and deoxy-Hb changes in 12 channels for each hemisphere.

In each hemisphere, the placement of the probes covered the temporal areas centered at T5 and T6 according to the International 10-20 system [27]. This was the same region examined in our other recent NIRS studies [2-4,6-9,14,26]. When the probes were positioned, the experimenter checked to see if the fibers were touching each infant’s scalp correctly. The Hitachi ETG-4000 system automatically detects whether the contact is adequate to measure the emerging photons for each channel. The channels were rejected from the analysis if adequate contact between the fibers and each infant’s scalp could not be achieved because of hair interference.

### Data analysis

Throughout the experiment, the infants’ behavior was recorded on videotape. As in our previous study [8,9], we removed the trials from analysis if (1) the infants did not look at the test stimuli for at least the first 7 s of the 10 s presentation or if they became fussy, (2) if the infants looked back at the face of experimenter during the preceding baseline period, or (3) if the trials included movement artifact which were detected by the analysis of sharp changes in the time series of the raw data of the NIRS.

The raw data of oxy-Hb, deoxy-Hb, and total-Hb from the individual channels were digitally high-pass-filtered at 0.02 Hz to remove any signal drift [1-4,6-9,26] and low-pass-filtered at 2.0 Hz in order to remove high frequently measurement noise. Then the raw data of each channel were averaged across the trials within a subject in a time series of 0.1 s time resolutions from 2 s before the test trial onset to 10 s after the test trial offset.

Based on the mean concentration of the time series, the Z-scores were calculated separately for oxy-Hb, deoxy-Hb, and total-Hb in both the *same-face* and *different-face* conditions for each channel within a subject. The Z-scores were calculated as the difference of the means of the baseline and test condition divided by the SD of the baseline using the following formula:

$$d=\left(M\mathrm{test}–M\mathrm{baseline}\right)/S$$

Accordingly, *M* test represents the averaged value of the raw data during the test trials (*different-face* and *same-face* condition) and *M* baseline represents that of the raw data during the vegetables baseline period. *S* represents the SD of the baseline. Although the raw data of NIRS were originally relative values and could not be averaged directly across subjects or channels, the normalized data such as the Z-scores could be averaged regardless of the unit [28,29].

As in Kobayashi et al. [8,9], we performed a statistical analysis against the mean Z-scores from 3 to 7 s after the face stimulus onset in each age group for oxy-Hb and deoxy-Hb, respectively. A two-tailed one-sample *t*-test against a chance level of 0 was conducted for the mean Z-score during the 3 to 7 s of the test trials in both temporal areas. Furthermore, for all 24 channels, each channel’s activation of oxy- and deoxy-Hb to both the *same-face* and *different-face* conditions was tested by a two-tailed one-sample *t*-test against the baseline. To eliminate the risk of a Type I error, we performed the corrections using the false discovery rate (FDR) [30]. We selected the channels that this statistical analysis revealed as having a significant increase as Channels of Interest (COI). Finally, we conducted a repeated measures ANOVA with two factors, condition and channels on the COI region.

## Competing interests

The authors declare that the research was conducted in the absence of any commercial or financial relationships that could be construed as a potential conflict of interests.

## Authors’ contributions

MK performed the experiment and analyses and wrote first draft of the manuscript. YO co-designed the experiment and manipulated the images of facial stimuli. SK, MKY and RK contributed to the design and revised the manuscript. All authors read and approved the final manuscript.

## Authors’ information

Yumiko Otsuka is now at School of Psychology, UNSW Australia, Sydney, Australia.
